# Brain abscess due to listeria monocytogenes: A case report and literature review

**DOI:** 10.1097/MD.0000000000026839

**Published:** 2021-08-06

**Authors:** Junliang Zhang, Shuangfeng Huang, Luyao Xu, Manli Tao, Yujun Zhao, Zhigang Liang

**Affiliations:** a Department of Neurology, Yantai Yuhuangding Hostipal Affiliated to Qingdao University; b Binzhou Medical University, Yantai, Shandong, China.

**Keywords:** bacteriological examination, brain abscess, listeria monocytogenes, neuroradiological examination

## Abstract

**Rationale:**

Listeria monocytogenes infective encephalitis is a rare phenomenon, which is more common in people with changed eating habits and immunodeficiency. To the best of our knowledge, listeria brain abscess is even more rare. In this case report, we summarized the clinical characteristics of listeria brain abscess, in order to explore the diagnosis and treatment of Listeria brain abscess, and raise awareness and attention to the disease.

**Patient concerns:**

A 64-years-old female patient presented to our institution with 4 days of right arm and leg weakness, the salient past history of the patient was nephrotic syndrome, membranous nephropathy diagnosed 6 months prior, for which she was prescribed glucocorticoids and cyclophosphamide.

**Diagnosis:**

Listeria monocytogenes was cultured in the blood of the patient. Comprehensive medical history and imaging features, she was diagnosed as listeria brain abscess.

**Interventions:**

The patient underwent ampicillin combined with meropenem but not surgery.

**Outcomes:**

The patient recovered without complications. At a 3-month follow-up visit, the condition was better than that before treatment.

**Lessons:**

Listeria brain abscess is an unusual form of listeriosis, its clinical manifestations lack specificity. Early accurate diagnosis and standardized treatment can effectively promote the recovery of neurological function as well as reduce the morbidity and mortality and improve the prognosis.

## 1 Introduction

Listeria monocytogenes is a gram-positive bacteria that enters the host body mainly through the intestinal tract, and causes listeriosis by way of food-borne infection.^[1]^ Among listeriosis, listeria brain abscess is very rare, accounting for about 10% of listeria central nervous system infections, and its clinical manifestations lack specificity.^[2]^ The incidence of central nervous system (CNS) listeriosis has increased due to the increased need for immunosuppressive drugs for organ and bone marrow transplantation.^[3]^ Listeria brain abscesses is now more often described and is associated with a high rate of concomitantb acteremia. But the neurologic outcomes have not been clearly defined for this form of listerial infection.^[4]^ We descripted a case of listeria brain abscess and reviewed the literature. From 1968 to 2020, only 84 cases were reported in the literature, brain MRI and bacteriological examination can help confirm the diagnosis. We combined the previous literature to review and analyze, hoping to increase people's awareness and attention to the disease.

## 2 Case presentation

**Figure 1:**
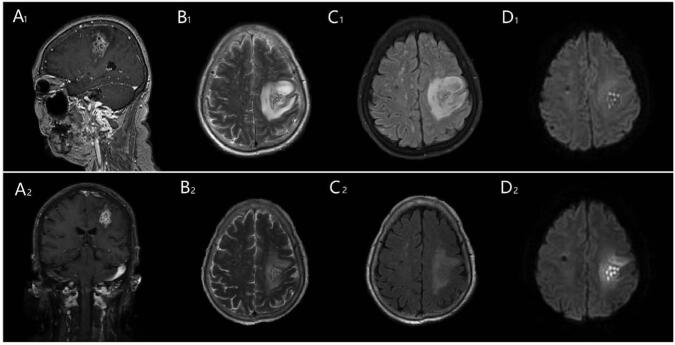
Longer signal on T1-weighted images on frontal parietal region of sagittal position and longer signal on T2-weighted images in the left frontoparietal junction area. (A1 and B1). Hyperintense in FLAIR and DWI sequence (C1 and D1). Multiple high signals with cluster distribution can be seen in the lesion area, with surrounding vasogenic edema and local mass effect. The same lesion area was reduced and edema was alleviated (A_2_–D_2_).

A 64-years-old female patient presented to our institution with 4 days of right arm and leg weakness, which progressed to hemiplegia of the right upper limb, and the right lower limb could not walk independently. Prior to presentation, the patient had no headache, dizziness, fever, and diarrhea. She denied consumption of unpasteurized cheese or changes in diet. Her medical history was significant with hypertension and diabetes for 10 years, and nephrotic syndrome, membranous nephropathy diagnosed 6 months prior, for which she was prescribed glucocorticoids and cyclophosphamide. Neurological examination found that the patient had a clear consciousness, the right side of the nasolabial sulcus was shallow, and the tongue was biased towards the right side while stretching. The muscle tension and muscle strength of the left limb were normal, but the muscle tension of the right limb was slightly lower, and the muscle strength of the right upper limb was grade 0, the muscle strength of the right lower limb was grade 4. The pathological sign of the left side was (−) and that of the right side was (+), without signs of meningeal irritation. National Institutes of Health Stroke Scale (NIHSS) score was 7 points. After admission, she was given treatment such as improving circulation, butylphthalide alleviates demyelinationand improves cognitive function by promoting mitochondrial dynamics, and reducing intracranial pressure. The patient's condition did not improve obviously, and there was fever, the highest temperature was 38.9°C. Brain enhanced MRI revealed an irregular, well-defined lesion within the left frontoparietal junction. (Longer signal on T1-weighted images on frontal parietal region of sagittal position and longer signal on T2-weighted images in the left frontoparietal junction area) (Fig. 1, A_1_, B_1_). (Hyperintense in FLAIR and DWI sequence) (Fig. 1, C_1_, D_1_). Multiple high signals with cluster distribution can be seen in the lesion area, with surrounding vasogenic edema enhancement and local mass effect. Comprehensive medical history and imaging features, considering the possibility of intracranial infection, she was started on empiric antimicrobial coverage with ceftriaxone for a suspected brain abscess. During the course of hospitalization, lumbar puncture showed cerebrospinal fluid pressure 135 mm H_2_O, white blood cells 13∗10^6/L, single cell nucleus 92.3%, glucose 5.52 mmol/L, chlorine 126.5 mmol/L; cerebrospinal fluid protein 443.0 mg/L. Generallaboratory values were unremarkable. The amino-terminal polypeptide of bacteriolysin O is a more specific antigen, and the serological assays developed from it may have more clinical diagnostic value. In this way, listeria monocytogenes was cultured in the blood of the patient. So she was diagnosed as listeria brain abscess, and the treatment regimen was changed to ampicillin (given by injection are 2 g every 6 h intramuscularly) combined with meropenem (given by injection are 2 g every 8 h intramuscularly). The patient recovered without complications, the temperature returned to normal and a post-blood culture showed listeria monocytogenes (−), she continued to take ampicillin for 3 weeks. The second brain enhanced MRI revealed the lesion area was reduced and edema was alleviated. (Fig. 1, A_2_–D_2_). Ampicillin was continued for a total of 42 days. At 3-month follow-up visit, she reported significant improvement in strength in his affected limbs extremities, however still required assistance with household activities.

## 3 Discussion

Listeria monocytogenes is a rare cause of brain abscess, which can cross the blood–brain or blood–choroidal barrier, invade endothelial cells through extracellular blood transmission, or retrograde migrate to the brain through nerve axons, and invading the CNS. Some authors propose axonal invasion as the pathological substrate of the characteristic multi-tubular appearance of listeria brain abscess referred as “tunnel-sign” or “worm-like” appearance.^[5]^ Reportedly, by way of the middle cerebral artery, listeria monocytogenes-infected macrophages may move through endothelial cells causing cerebritis which leads to brain abscess formation.^[6]^ Because most patients with multiple abscesses in the brain present a unique phenomenon of distribution along white matter fibers on imaging, some scholars believe that Listeria monocytogenes can be transmitted through white matter fibers in the CNS.^[7]^ The route of infection in this patient may be blood-borne because of the positive blood culture. In combination with the brain abscess of the patient, it is distributed along the white matter fibers, so it is speculated that Listeria monocytogenes enters the CNS through the blood and then spreads through the white matter fibers.

Individuals with impaired cell-mediated immunity are at risk of developing listerial infections. The majority of human infections occur in newborns, immunosuppressants, the weak and the elderly.^[8,9]^ Commonly, listeria monocytogenes central nervous system infection presents as meningitis, but very rarely, it can also lead to brain abscesses, and such patients usually have a poor prognosis. Listeria brain abscess was first reported in humans as a bloodstream infection in a 17-year-old boy.^[10,11]^ The reported incidence of CNS listeriosis has increased with the increase in the frequency of immunosuppressive drugs required for organ and bone marrow transplantation. We describe a case of brain abscess due to listeria monocytogenes and discuss them by reviewing the literature on this topic. To the best of our knowledge, only 84 cases were reported in the literature from 1968 to 2020. Statistical patient information: the mean age of the patients was 52.9,52 fifty-two of these patients were male (61.9%). The mortality rate was 25.0%. Fifty-five out of 84 had one or more risk factors for the development of listeria brain abscess (65.5%). The probability of isolating listeria monocytogenes from cerebrospinal fluid or brain abscess and blood culture is 69.1%. Forty out of 84 patients received a monotherapy regimen (47.6%), ampicillin was the most commonly prescribed antibiotic, while a combination therapy was prescribed for 32/84 (38.1%) cases, there was no significant difference in mortality between the two groups. The treatment regimen of 8 patients is unknown. One out of four patients was died who received brain abscess drainage combined with drug (Table 1).

**Table 1:** Summary of reported cases with Listeria brain abscess.

		Treatment		
High-risk patients	Cultivation rate	Monotherapy	Combination therapy	Surgery + drugs
55/84 (65.6%)	58/84 (69.1%)	40/84 (47.6%)	32/84 (38.1%)	4/84 (4.8%)

At present, there are no definitive guidelines for the diagnosis and treatment of listeria monocytogenes brain abscess. Combined with the previous literature, we propose the following five points to consider the diagnosis of listeria brain abscess:

1. The form of the disease is acute onset, most of the patients with low immune function or changes in eating habits, and may have a history of prodromal infection.
2. Fever is the most common first symptom in clinical manifestations, which can be accompanied by headache, nausea, meningeal irritation, and focal neurological dysfunction.
3. MRI showed longer signal on T2-weighted images, and hyperintense in DWI images. Rim enhancement, perifocal edema, and local mass effect are the typical characteristics of brain abscess.
4. Excluding intracranial space occupying and other bacterial brain abscesses.
5. Listeria monocytogenes was cultured in the blood or cerebrospinal fluid of the patient.

To the best of our knowledge, we found that listeria brain abscess often affects the brain stem, usually unilateral, and the most severe areas are medulla oblongata and pons, followed by supratentorial white matter and cerebellar hemispheres.^[12]^ Blood cultures are positive in 61% with repeated cultures, but results are obtained with a significant delay.^[13]^ Analysis of cerebrospinal fluid samples show absence of pleycotosis in 22% and negative cultures in 59%, while Gram's stain has a diagnostic yield of less than 4%.^[14]^ Due to the presence of bacterial cells and flagella antigens, people can establish methods based on antigen–antibody reactions, such as enzyme-linked immunoassay (ELISA) to detect pathogenic bacteria in specimens. In recent years, nucleic acid molecular hybridization technology and polymerase chain reaction (PCR) methods have been used for clinical diagnosis with strong sensitivity. But the positive samples of molecular testing still need to be referenced by traditional methods. The treatment of listeria brain abscess is similar to that of other brain abscesses. Sensitive antibiotics should be given and the focus should be removed in time. The first choice is ampicillin combined with gentamicin, which is more effective than penicillin combined with gentamicin.^[15]^ There are also literatures showing that ampicillin alone did not lead to an increase in mortality. Meropenem has also been successfully used to treat the infection, we used ampicillin combined with meropenem in our case and achieved good prognosis. Patients with high suspicion and diagnosis should be treated with ampicillin or penicillin for 2 to 4 weeks. After the body temperature returns to normal, it should be treated continuously for at least 1 week. For those with low immunity, the course of treatment can be extended to 6 weeks. Those who have no response, recurrence, large abscess or infection with drug-resistant microorganisms should be timely surgical drainage or surgical resection. Surgical aspiration is suggested for large abscesses (>2.5 cm) located in deep brain matter (e.g., cerebellum and diencephalon).^[16]^ It has been reported that hyperbaric oxygen therapy can effectively improve the prognosis of patients with inoperable deep brain abscess.^[17]^ A review of listeria brain abscesses showed that patients with surgical drainage either through stereotactic aspiration or craniotomy had a higher suevival rate than patients treated with only antibiotics.^[3]^ In the present case, the lesion involved the left frontoparietal junction, given the patient was sensitivity to ampicillin, we didn’t have an operation. In addition, some researchers believe that T-cell activation, complement activation, interferon and endothelial growth factor production are important in the immune response to listeria monocytogenes,^[18]^ and may affect the outcome, so immunomodulatory therapy may be a new breakthrough in the treatment of listeria monocytogenes infection. Brain abscess due to listeria has a poor prognosis and is associated with elevated mortality.^[19]^ Mortality resulting from listerial brain abscess, whether solitary or multiple, is nearly three times higher than nonlisterial brain abscess, probably in part because of both underlying diseases and immunosuppressive therapy.^[7]^

## 4 Conclusion

Listeria brain abscess is an unusual form of listeriosis, which can infect the meninges, also invade the brain parenchyma, its clinical manifestations lack specificity. Ampicillin is the most commonly used sensitive antibiotic, but the theraphutic regime should be individualized, early accurate diagnosis and standardized treatment can effectively promote the recovery of neurological function as well as reduce the morbidity and mortality and improve the prognosis.

## Acknowledgments

All clinical diagnoses and treatments in this article were in accordance with China's national guidelines for diagnosis and treatment and in compliance with the requirements of the Hospital Ethics Committee. Patients were asked to sign informed consent before participation. Written informed consent was obtained from the individual(s) for the publication of any potentially identifiable data included in this article.

## Author contributions

Junliang Zhang, Shuangfeng Huang, Luyao Xu, Manli Tao and Yujun Zhao involved in data collection and clinical care of this patient. Junliang Zhang drafted the manuscript, Zhigang Liang provided technical and material support, helped design the study. All authors contributed to the article and approved the submitted version.

**Data curation:** Junliang Zhang, Shuangfeng Huang, Luyao Xu, Manli Tao.

**Methodology:** Zhigang Liang.

**Software:** Junliang Zhang.

**Supervision:** Zhigang Liang, Yujun Zhao.

**Writing – original draft:** Junliang Zhang.

**Writing – review & editing:** Zhigang Liang.
